# The association between sleep quality and psychological distress among older Chinese adults: a moderated mediation model

**DOI:** 10.1186/s12877-021-02711-y

**Published:** 2022-01-10

**Authors:** Chichen Zhang, Shujuan Xiao, Huang Lin, Lei Shi, Xiao Zheng, Yaqing Xue, Fang Dong, Jiachi Zhang, Benli Xue

**Affiliations:** 1 School of Health Management, Southern Medical University, No.1023 Shatai South Road, Baiyun District, Guangzhou, 510515 Guangdong China; 2 Department of Health Management, Nanfang Hospital, Southern Medical University, Guangzhou, Guangdong China; 3 Institute of Health Management, Southern Medical University, Guangzhou, Guangdong China; 4 Shool of Public Health, Southern Medical University, Guangzhou, Guangdong China; 5 The First School of Clinical Medicine, Southern Medical University, Guangzhou, Guangdong China

**Keywords:** Sleep quality, Activities of daily living, Physical activity, Psychological distress, Moderated mediation

## Abstract

**Background:**

Previous research has found a link between sleep quality and psychological distress. However, the underlying mechanisms of this connection have still not been well explored. The aim of this study was to examine the roles of activities of daily living (ADL), physical activity, and perceived social support in the link between sleep quality and psychological distress among older adults.

**Methods:**

Three thousand two hundred fifty valid individuals (aged 60 years or above) participated in face-to-face questionnaire-based surveys. Older individuals were assessed using the Pittsburgh Sleep Quality Index, Barthel Index, International Physical Activity Questionnaire, Perceived Social Support Scale, and Depression Anxiety Stress Scale-21. The PROCESS macro developed by Hayes was used to conduct moderated mediation analysis.

**Results:**

In older adults, sleep quality had a substantial direct influence on psychological distress. ADL mediated the association between sleep quality and psychological distress. Physical activity significantly reduced ADL’s mediating influence on the relationship between sleep quality and psychological distress. Furthermore, perceived social support moderated the direct relationship between sleep quality and psychological distress, as well as the second half of the mediating effect of the ADL. Moreover, physical activity and perceived social support were preventive factors that might successfully mitigate the detrimental effects of poor sleep quality on ADL and psychological distress.

**Conclusions:**

The findings contribute to existing understanding by clarifying the fundamental mechanisms that link sleep quality and psychological distress. These results may provide a valuable reference to the Chinese government for improving mental health in older individuals.

## Background

Globally, the aging population is quickly growing, particularly in China. With the accelerated population aging, more attention must be paid to the health issues of older adults [1]. Psychological distress would be a more practical concept in the context of comprehensive mental disorder when considering public health strategies [2]. Psychological distress is largely defined as a state of emotional suffering, typically characterized by depressive and anxiety symptoms [3], which was assessed by the Depression, Anxiety, and Stress Scale (DASS-21) in the current study [4]. According to the World Health Organization (WHO), depression is a prevalent mental disease that affects around 350 million people worldwide [5]. A study found that 8.7% of old adults had both depressive and anxious symptoms in China [6]. Psychological distress is a significant public health issue because it causes cognitive impairment, functional decline, physical disability, and increased health-care usage [7, 8]. Psychological distress has a detrimental influence not only on one’s physical health but also on one’s quality of life [9]. Thus, it is critical to determine the correlates of psychological distress in order to theoretically and practically avoid the development of psychological distress and achieve healthy aging.

Sleep, a changeable lifestyle habit, has received special study due to its importance in maintaining good mental and physical health [10]. However, accompanied by health conditions related to old age, this exacerbates the frequent sleep problems among older adults. The overall prevalence rates of poor sleep quality were 33.8% among Chinese older adults living in a rural area [11]. Previous studies in China suggest that sleep interruption is associated with anxiety and depressive symptoms [12, 13]. A prior study found that elderly women with poor sleep quality also had poor mental health [14]. These findings suggest that sleep quality was associated with psychological distress in older adults. The growing prevalence of sleep problems in old age, as well as the associated health risks, has spurred a rise in research and policy discussion. Although a link was identified between sleep quality and psychological distress, the specific mechanistic mechanisms behind this link remain unknown. Exploring the moderated mediation pathways between sleep quality and psychological distress contributes to the development of more effective methods of early diagnosis and prevention of psychological distress.

Previous research has indicated that found that psychological distress was significantly correlated with ADL among Chinese older adults [15]. ADL is another factor associated with psychological distress. Disabled older individuals are more vulnerable to mental health problems than non-disabled older adults. Previous studies have also discovered that poor sleep quality was associated with functional limitations in older adults [16, 17]. This may be that the quality of sleep affects the state of cognitive function and ultimately affects the ability to perform daily living activities. However, research on the association between sleep quality, ADL, and psychological distress has been scarce. According to the findings, ADL may act as a mediator between poor sleep quality and psychological distress.

Physical activity is another significant modifiable lifestyle element that contributes to better health. Regular physical activity in older individuals can enhance overall health and restore functional abilities (balance, maximum aerobic power, peak muscular force, and flexibility) [18]. The beneficial impact of physical activity on overall health has been extensively documented in the previous literature [19, 20]. Indeed, varied levels of engagement in regular physical activity programs among older individuals have the ability to buffer, postpone, or counteract anticipated functional decline, allowing those affected to maintain the highest level of quality of life [21]. Physical activity has been shown to moderate the connection between chronic disease and functional limitations [22]. A longitudinal decline in physical function and disability is moderated most notably by physical activity [23]. It is uncertain, however, if physical activity has a moderating effect on the influence of sleep quality on ADL in older individuals.

Perceived social support refers to the emotional experience and pleasure that individuals receive from society when they are appreciated, supported, and understood [24]. According to the stress buffering theory, social support can give resources to mitigate the negative impact of stress and problems on health, thus maintaining and improving an individual’s health outcomes [25, 26]. Previous research has suggested that social support is adversely related to psychological distress [27, 28]. When individuals experience psychological distress, social support can be considered to be a coping strategy [29]. As a result, older individuals with sleep problems or functional impairments may have lower levels of psychological distress if they have enough social support. However, little research has been conducted to investigate whether social support moderates the connections between sleep quality and psychological distress in older individuals.

**Fig. 1:**
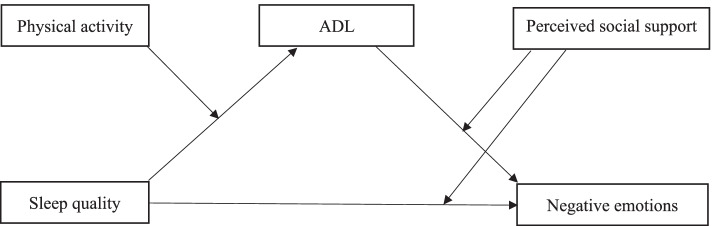
Hypothesized model

As a result, the current study will use a moderated mediation analysis to explore the link between sleep quality, ADL, perceived social support, and psychological distress. Given the severity of the mental issue, the current study began with the following hypotheses: 1) The association between poor sleep quality and psychological distress may be mediated by functional limitations. 2) The mediation effect of ADL would be moderated by physical activity. That is, older individuals who engage in more physical activity would be shielded from the detrimental effects of poor sleep quality by improving their function level. 3) Perceived social support would attenuate the direct and indirect effect of sleep quality on psychological distress. The proposed moderated mediation model in this present study is depicted in Fig. 1.

## Materials and methods

### Participants and ethics statement

A cross-sectional questionnaire study was carried out in Shanxi Province, Central China, which consists of 11 cities. In order to obtain a representative sample of older adults, we utilized a multi-stage stratified cluster sampling approach to choose individuals aged 60 and above in 11 cities, which was explained in detail elsewhere [1]. Finally, we chose older adults at random who met the study’s criteria. The inclusion criteria for this study were: (1) being aged 60 and above and (2) having clear awareness and barrier-free communication skills. Those who had difficulty communicating were excluded.

The Ethics Committee of Shanxi Medical University approved all study methods. All participants were informed about the study’s goal and signed an informed consent form. The study was conducted from June to August 2019. A total of 3266 questionnaires were issued, with 3250 respondents effectively completing the surveys, resulting in a 99.51% effective response rate.

### Materials

#### Pittsburgh sleep quality index (PSQI)

The validated PSQI was used by older individuals to measure the quality of their sleep over the past 1 month [30]. PSQI includes 19 items that assess seven domains: subjective sleep quality, sleep latency, sleep duration, habitual sleep efficiency, sleep disturbances, use of sleep medication, and daytime dysfunction over the last month [11]. Each component is scored from 0 to 3. The PSQI score ranges from 0 to 21, with the higher the score, the worse the quality of sleep. In the study, the Cronbach’s α for PSQI was 0.758.

#### Barthel index

In this study, the Barthel Index consists of ten items for assessing ADL [31]. This index comprises 10 items: self-feeding, self-bathing, grooming, getting dressed, bowel control, bladder control, using the toilet, chair/bed transfer, mobility, and climbing stairs. Each item is scored proportionally and a given number of points are assigned to each level or rank [32]. The total score is 100 points; the higher the score, the better the daily living abilities [33]. The ADL scale has been reported to have high validity and reliability in the Chinese older population [34]. In this sample, the ADL Cronbach’s α was 0.937.

#### International physical activity questionnaire (IPAQ)

The Chinese version of the International Physical Activity Questionnaire long-form (IPAQ) was utilized in the study to measure the physical activity levels of older individuals. The IPAQ consists of 27 questions that reflect on the previous 7 days’ activities [35]. According to the Chinese data processing and analysis standards for the IPAQ [36], in the current study, there are three levels of physical activity: low, moderate, and high. In this study, there were 723 (22.24%) older adults in the low physical activity group, 1321 (40.65%) older adults in the moderate physical activity group, and 1206 (37.11%) older adults in the high physical activity group.

#### Perceived social support scale (PSSS)

The Perceived Social Support Scale (PSSS) was used to assess the quality of social support received from three different sources: family, friends, and significant others [37]. The perceived social support scale (PSSS) is a 12-item self-reported inventory. Participants were asked to assess their level of agreement with each topic on a 7-point Likert scale ranging from 1 (strongly disagree) to 7 (strongly agree) (very strongly agree). The items were added together to get total scores ranging from 12 to 84, with higher scores indicating higher levels of perceived support. Among older adults, the scale has been frequently used and has strong psychometric characteristics [38]. Cronbach’s α for the whole scale was 0.968 in the current research.

#### Depression, anxiety, and stress scale (DASS-21)

The Chinese version of the Depression, Anxiety and Stress Scale (DASS-21) was used to measure psychological distress in this sample [39]. Each item is scored on a 4-point Likert scale (0 = did not apply to me at all and 3 = applied to me very much), with a higher total score indicating more severe levels of distress [40]. For each subscale, the summed items were multiplied by two to represent the longer version of the questionnaire, DASS-42. Scores on the DASS-21 range from 0 to 126. As with the previous measures, the Chinese version of this scale has been used in many studies with high validity and reliability [39, 41]. The Cronbach’s α for DASS-21 was 0.950 in the studied sample, showing excellent internal consistency in the Chinese older population.

#### Control variables

On the basis of existing research, we identified possible confounders for psychological distress [42, 43]. Sex, age, residence, education level, monthly income, marital status, chronic disease status, smoking and drinking status are all possible confounders. Self-reported data on chronic illnesses was supplemented with diagnostic evidence from medical records or physician prescriptions.

### Statistical analysis

Data was analyzed using SPSS Version 24.0 (IBM, Armonk, NY, USA). The association between the different continuous variables was examined using Pearson’s correlation. Both single mediation (model 4) and moderated mediation (model 28) analyses were based on bootstrapping (5000 bootstrap samples) using 95% confidence intervals. Sex, residence, age, education level, monthly income, marital status, smoking status, drinking status, and chronic conditions were included as covariates. ANOVA was used to compare differences in multimorbidity, depression, anxiety, and stress scores, with results considered statistically significant when *p*-values were less than 0.1. The covariates were factors that were statistically significant in univariate analysis: sleep quality as an independent variable (X), ADL as the M variable, physical activity as the W variable, perceived social support as the Z variable, and psychological distress as the dependent variable (Y). The macro calculated and tested direct effects, total effects, and indirect effects. Our computations were carried out in two phases, based on the bootstrap moderating and mediation effect tests.

Harman’s single factor test was employed in the study to uncover common technique bias. In common approach bias testing, this method has been frequently utilized [44, 45]. Following the principal component analysis, 16 eigenvalues larger than 1 were recovered. The first component described the general variance of all research variables, which was caused by common technique biases and relationships between research variables. The first component that explained the variation was 21.354%, which was much lower than the crucial value of 40% [45]. As a result, we determined that the current study has no significant common method biases.

## Results

### Socio-demographic characteristics

Of the 3250 participants, 1515 (46.62%) were male and 1735 (53.38%) were female. The majority of the older people were 60–69 years old (54.43%), 1797 (55.29%) lived in rural areas and 1453 (44.71%) in urban areas. 76.37% of the older adults were married.

### Correlation analysis

Table 1 shows the correlation matrix for the main research variables. Prominently, both PSQI score and DASS-21 score were negatively correlated with Barthel Index score (for PSQI score, *r* = − 0.263, *P* < 0.001; for DASS-21 score, *r* = − 0.473, *P* < 0.001) and physical activity level (for PSQI score, *r* = − 0.100, *P* < 0.001; for DASS-21 score, *r* = − 0.217, *P* < 0.001). The PSQI score was significantly and positively correlated with psychological distress (*r* = 0.444, *P* < 0.001), and the Barthel Index score was significantly and positively correlated with physical activity level (*r* = 0.364, *P* < 0.001) and PSSS score (*r* = 0.237, *P* < 0.001). The PSSS score was negatively correlated with the PSQI score (*r* = − 0.180, *P* < 0.01) and the DASS-21 score (*r* = − 0.320, *P* < 0.001). The PSQI score was negatively correlated with physical activity level (*r* = − 0.100, *P* < 0.001).

**Table 1:** Descriptive statistics and correlations among the key variables (*n* = 3250)

Variables	M ± SD	1	2	3	4	5
1 PSQI score	5.082 ± 3.436	1				
2 Barthel Index score	91.699 ± 18.350	−0.263**	1			
3 Physical activity level	2.149 ± 0.756	−0.100**	0.364**	1		
4 PSSS	66.155 ± 13.687	−0.180**	0.237**	0.228**	1	
5 DASS-21 score	19.142 ± 20.566	0.444 **	−0.473**	−0.217**	− 0.320**	1

Note: **p < 0.01, *PSSS* Perceived Social Scale score

### Testing the mediation model

After adjusting for factors including sex, residence, age, education level, monthly income, marital status, smoking status, drinking status, and chronic conditions, the significance of the direct, indirect, and total effects in a mediation model was determined using PROCESS (model 4). According to the findings (Table 2), poor sleep quality was positively associated to the level of psychological distress in older adults (*B* = 2.499, *t* = 25.345, *P* < 0.001). After adding the Barthel Index score as a mediating variable, both psychological distress (*B* = 1.950, *t* = 21.074, *P* < 0.001) and the Barthel Index (*B* = − 1.270, *t* = − 13.610, *P* < 0.001) were found to be positively and negatively associated with poor sleep quality. Moreover, the Barthel Index score was a negative predictor of psychological distress (*B* = − 0.433, *t* = − 25.542, *P* < 0.001). On bias-corrected percentile bootstrap analysis, the mediation effect of ADL on the relationship between sleep quality and psychological distress was significant (ab = 0.550, 95% CI 0.433, 0.671), and accounted for 22.01% of the total effect. Therefore, ADL mediated the relationship between sleep quality and psychological distress to some extent.

**Table 2:** Testing ADL as a mediator in the relationship between poor sleep quality and psychological distress

Model pathways	R^2^	*B*	SE	t	95%CI
Sleep quality→Psychological distress	0.213	2.499	0.099	25.345	(2.306, 2.692)
Sleep quality→ADL	0.115	−1.270	0.093	−13.610	(− 1.453, − 1.087)
Sleep quality→Psychological distress	0.345	1.950	0.093	21.074	(1.768, 2.131)
ADL → Psychological distress		−0.433	0.017	−25.542	(−0.466, − 0.400)

Note: Covariates used to control included sex, residence, age, education level, monthly income, marital status, smoking status, drinking status, and chronic conditions. *B* regression coefficient, *SE* standard error

### Testing the moderated mediation model

After adjusting for factors including sex, residence, age, education level, monthly income, marital status, smoking status, drinking status, and chronic conditions, we utilized Hayes’ PROCESS to run the moderated mediation model using model 28 as a last check. The moderated mediation analysis (Table 3) indicated that poor sleep quality was negatively associated with ADL (*B* = − 1.094, *t* = − 12.486, *P* < 0.001). Moreover, both poor sleep quality (*B* = 1.762, *t* = 19.332, *P* < 0.001) and ADL (*B* = − 0.397, *t* = − 20.086, *P* < 0.001) were positively and negatively related to psychological distress. Perceived social support was negatively associated with psychological distress (*B* = − 0.286, *t* = − 13.013, *P* < 0.001). The interaction effect of sleep quality and physical activity on ADL was significant (*B* = 1.015, *t* = 9.607, *P* < 0.01), which indicates that the association between sleep quality and ADL was moderated by physical activity. Whereas, the interaction effect of ADL and perceived social support on psychological distress was not significant (*B* = − 0.001, *t* = − 1.095, *P* = 0.274). The interaction effect of sleep quality and perceived social support on psychological distress was also significant (*B* = − 0.033, *t* = − 5.280, *P* < 0.001), which indicates that perceived social support reduced the association between sleep quality and psychological distress.

**Table 3:** Testing ADL as a mediator and physical activity and perceived social support as two moderators in the relationship between sleep quality and psychological distress

Model pathways	R^2^	*B*	SE	*t*	95%CI
Sleep quality→ADL	0.226	−1.094	0.088	−12.486	(− 1.266, −0.923)
Physical activity→ADL		7.421	0.388	19.130	(6.660, 8.181)
Sleep quality×Physical activity→ADL		1.015	0.106	9.607	(0.808, 1.222)
Sleep quality→Psychological distress	0.380	1.762	0.091	19.332	(1.583, 1.940)
ADL → Psychological distress		−0.397	0.020	−20.086	(−0.436, − 0.358)
PSSS→Psychological distress		−0.286	0.022	−13.013	(−0.329, − 0.243)
Sleep quality×PSSS→Psychological distress		−0.033	0.006	−5.280	(−0.046, − 0.021)
ADL × PSSS→Psychological distress		−0.001	0.001	−1.095	(−0.004, 0.001)

Note: Covariates used to control included sex, residence, age, education level, monthly income, marital status, smoking status, drinking status, and chronic conditions

**Fig. 2:**
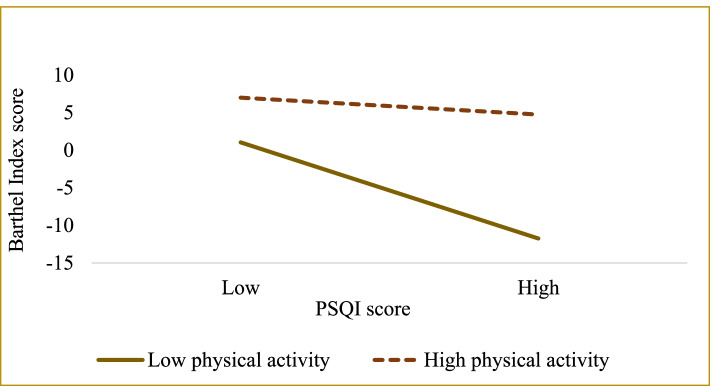
Moderation of physical activity to sleep quality and psychological distress

The results of the simple slope test (see Table 4 and Fig. 2) further suggest that when physical activity is low, poorer sleep quality is negatively associated with the Barthel Index score (*B*_simple_ = − 1.862, *t* = − 16.128, *P* < 0.001). However, in older adults with high physical activity, the association between PSQI score and Barthel Index score was still significant (*B*_simple_ = − 0.327, *t* = − 2.687, *P* = 0.007). These results indicate that the direct association between sleep quality and ADL became stronger with a decrease in physical activity. Thus, physical activity is a protective physiological component that can effectively mitigate the harmful consequences of poor sleep quality on psychological distress.

**Table 4:** The impact of sleep quality on ADL at different levels of physical activity

Physical activity	*B*	SE	*t*	*P*	Boot LLCI	Boot ULCI
1.393 (M-SD)	−1.862	0.115	−16.128	0.000	−2.088	−1.636
2.149 (M)	−1.094	0.088	−12.486	0.000	−1.266	−0.923
2.905 (M + SD)	−0.327	0.122	−2.687	0.007	−0.565	−0.088

Note: *LL* low limit, *CI* confidence interval, *UL* upper limit, *M* mean, *SD* standard deviation

**Fig. 3:**
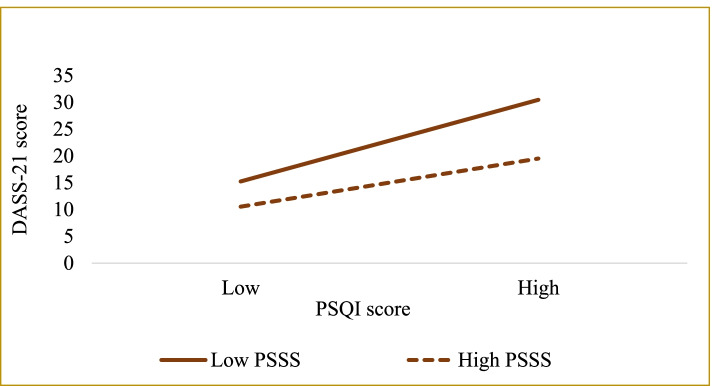
Moderation of perceived social support to sleep quality and psychological distress

The results of the simple slope test (see Table 5 and Fig. 3) further suggest that when social support is perceived to be low, poorer sleep quality is positively associated with greater psychological distress (*B*_simple_ = 2.217, *t* = 18.386, *P* < 0.001). In older adults with high perceived social support, the association between sleep quality and psychological distress was significant (*B*_simple_ = 1.306, *t* = 10.040, *P* < 0.001). These results indicate that the direct relationship between sleep quality and psychological distress has become weaker with an increase in perceived social support. Thus, perceived social support is a protective psychological component that can substantially mitigate the negative effects of poor sleep quality on psychological distress.

**Table 5:** The impact of sleep quality on psychological distress at different levels of perceived social support

Perceived social support	*B*	SE	*t*	*P*	Boot LLCI	Boot ULCI
52.468 (M-SD)	2.217	0.121	18.386	0.000	1.980	2.453
66.155 (M)	1.762	0.091	19.332	0.000	1.583	1.940
79.842 (M + SD)	1.306	0.130	10.040	0.000	1.051	1.562

Note: *LL* low limit, *CI* confidence interval, *UL* upper limit, *M* mean, *SD* standard deviation

**Fig. 4:**
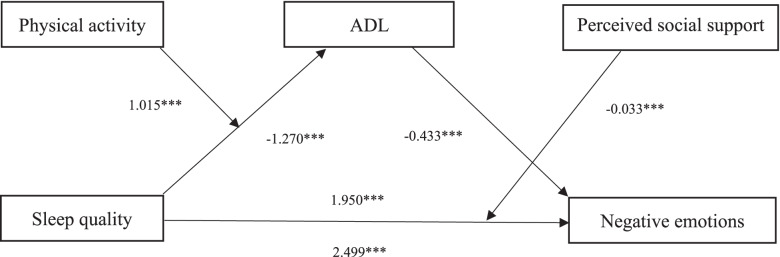
A moderated mediation model of the association between sleep quality and psychological distress through ADL, physical activity and perceived social support. Path coefficients are shown. Note: *p < 0.05, **p < 0.01, ***p < 0.001

Figure 4 illustrates the moderated mediation pathway model. The path coefficients revealed that all of the model’s relationships were significantly positive and negative. After including the mediator of Barthel Index score and moderators of physical activity and perceived social support, the direct effect of sleep quality on psychological distress was still significant. Therefore, the link between sleep quality and psychological distress was achieved partly through ADL and these associations were moderated by physical activity and perceived support.

## Discussion

In the present study, we discovered that the association between sleep quality and psychological distress was partially mediated by ADL among older individuals, indicating that poor sleep quality may have both a direct and indirect influence on psychological distress. Physical activity moderated the indirect impact, which was greater in older people with lower levels of physical activity. Furthermore, perceived social support reduced the direct and indirect effect, and the effect was greater in older individuals with lower levels of perceived social support. These findings contribute to elucidating the probable causes of psychological distress and facilitating tailored treatments for people aimed at improving the mental health of older adults.

This study revealed a significant correlation between sleep quality and psychological distress and older individuals with poor sleep quality were significantly related to severe psychological distress, which is consistent with previous research results [46]. Sleep quality had a significant influence on psychological distress. Our findings could be explained by the fact that poor sleep quality, such as difficulty falling asleep and short sleep duration, may worsen mood and increase anxiety with daytime fatigue, which can lead to an increase in psychological distress. Moreover, the relationship between poor sleep quality and depression and anxiety may be explained by an increased activation of REM sleep mechanisms [47].

Consistent with previous studies [12, 48], our mediation findings suggest that ADL had a strong mediating influence on the relationship between sleep quality and psychological distress. This study revealed that the worse older adults reported sleep quality, the greater degree of function restrictions of older adults, and hence the severity of psychological distress. One probable explanation is that poor sleep quality has a detrimental effect on both the physical and psychological elements of life. Older individuals who have ADL limitations are less likely to be able to care for and support themselves [46], leading to negative psychological experiences. Long-term negative psychological experiences, such as guilt and uselessness, seriously affected the mental health of older individuals [46]. These findings suggest that ADL may play an essential part in mediating the association between poor sleep quality and psychological distress. However, it merely mediates the relationship between poor sleep quality and psychological distress to a limited extent. This suggests that future research should explore additional mediators in order to offer a more comprehensive view of the complex link between poor sleep quality and psychological distress in older individuals. Although it is restricted to reaching a conclusion on the causative linkages based on our cross-sectional data, the current findings indicate poor sleep quality may confer a sensitivity to psychological distress by raising function restrictions levels, extending prior study findings. Furthermore, these findings have a significant practical implication: treatments for the prevention of psychological distress in older individuals would benefit from focusing more on assisting them in coping with physical impairment rather than only on relieving sleep problems.

The effect of physical activity on the relationship between sleep quality and ADL was also explored in this research. We found that physical activity moderated the association between sleep quality and ADL in Chinese older adults. Importantly, our analysis showed that not all older individuals with poor sleep quality are experiencing growing function limitations, increasing the likelihood of experiencing psychological distress. Physical activity was not only associated with ADL, but also moderated the strength of the mediating effect of ADL. Similarly, prior research has found a link between physical activity and physical functioning [23, 49]. Older individuals who engage in a lot of physical activity tend to be more protected against function decline and psychological distress. The result emphasized the need for early intervention through promotion of regular physical activity. Public health initiatives should concentrate on developing strategies to promote long-term regular physical activity by taking mental health status and functional status into account as conditions associated with physical activity.

According to our moderated mediation analysis, perceived social support moderated the direct relationship between sleep quality and psychological distress, as well as the second half of the mediating effect of the ADL. Previous studies have found a link between perceived social support and psychological distress [50, 51]. Higher perceived social support could be used to maintain a stable psychological state. Older individuals with higher levels of perceived social support have more psychological resources and are able to call on their psychological resources more effectively to buffer the negative effects of stress or adversity on their mental health. Another study further showed that receiving emotional support promoted parents’ psychological health among all combinations of support, and receiving all the three types of financial, instrumental and emotional support together raised their subjective wellbeing most [52]. In the current study, perceived social support was found to protect against the psychological distress of older adults. Firstly, for families, children should give more care to older adults, not only financial support but also emotional care. Secondly, communities need to build as many senior community activity centers as possible to promote communication and establish new social relationships among older adults. In addition, when giving social support to older adults, we should pay attention not only to the quantity of social support given, but also to the quality of social support given. That is, pay more attention to the older adult’s own feelings and improve their ability to perceive social support to really improve the psychological health of older adults.

## Limitations

The present study has several limitations in terms of interpreting results. Firstly, the cross-sectional data made making causal inferences between the identified variables and psychological distress challenging. In the future, further longitudinal studies need to be performed to investigate the causal link between sleep quality and psychological distress. Secondly, while we have accounted for certain confounders in our analysis, there may be residual confounding factors that we have not controlled for due to data availability, such as nutrition, some medications, and mild cognitive impairment.

## Conclusions

In conclusion, ADL partially mediated the link between sleep quality and psychological distress in older adults. Physical activity moderated the mediation effect, and the effect was stronger in older individuals who were less engaged. Moreover, perceived social support moderated the direct effect of sleep quality on psychological distress, as well as the second half of the mediating effect of the ADL, with a bigger effect found in older individuals with lower perceived social support than in those with higher perceived social support. To enhance psychological health, behavioral and physiological interventions targeting older adults are supposed to focus on early adoption and improve physical activity, ADL, and perceived social support among older adults in China.

## Data Availability

The datasets used and/or analyzed during the current study are available from the corresponding author (Prof. Chichen Zhang) on reasonable request.

## Abbreviations

ADL: Activities of daily living
PSSS: Perceived Social Scale score
LL: Low limit
CI: Confidence interval
UL: Upper limit
